# Lung Macrophage Phenotypes and Functional Responses: Role in the Pathogenesis of COPD

**DOI:** 10.3390/ijms19020582

**Published:** 2018-02-15

**Authors:** Kei Yamasaki, Stephan F. van Eeden

**Affiliations:** Centre for Heart Lung Innovation, Department of Medicine, University of British Columbia, Vancouver, BC V6Z1Y6, Canada; yamasaki@med.uoeh-u.ac.jp

**Keywords:** lung macrophage phenotypes, chronic obstructive pulmonary disease, phagocytic function of macrophage

## Abstract

Lung macrophages (LMs) are essential immune effector cells that are pivotal in both innate and adaptive immune responses to inhaled foreign matter. They either reside within the airways and lung tissues (from early life) or are derived from blood monocytes. Similar to macrophages in other organs and tissues, LMs have natural plasticity and can change phenotype and function depending largely on the microenvironment they reside in. Phenotype changes in lung tissue macrophages have been implicated in chronic inflammatory responses and disease progression of various chronic lung diseases, including Chronic Obstructive Pulmonary Disease (COPD). LMs have a wide variety of functional properties that include phagocytosis (inorganic particulate matter and organic particles, such as viruses/bacteria/fungi), the processing of phagocytosed material, and the production of signaling mediators. Functioning as janitors of the airways, they also play a key role in removing dead and dying cells, as well as cell debris (efferocytic functions). We herein review changes in LM phenotypes during chronic lung disease, focusing on COPD, as well as changes in their functional properties as a result of such shifts. Targeting molecular pathways involved in LM phenotypic shifts could potentially allow for future targeted therapeutic interventions in several diseases, such as COPD.

## 1. Introduction

**Figure 1:**
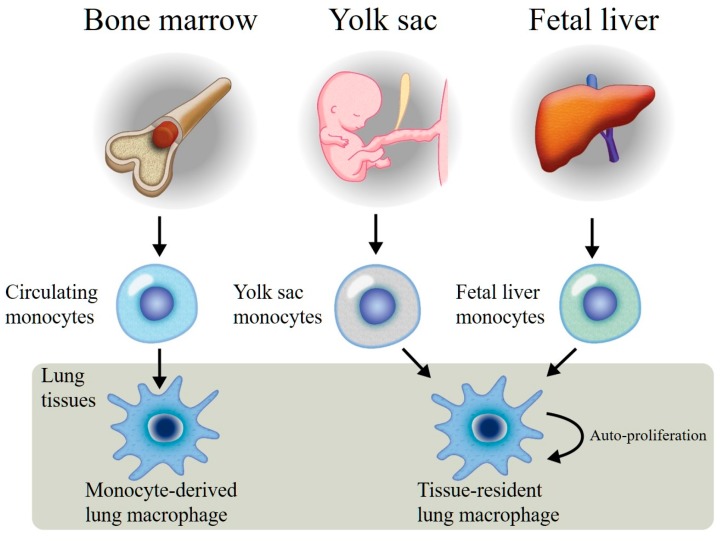
Monocyte-derived lung macrophages originating from bone marrow pluripotential stem cells, released into the circulating blood, and recruited into the lung tissues. These macrophages have limited proliferation functions. Tissue-resident lung macrophages are generated from both the yolk sac and fetal liver, recruited into the lungs during the early stage of lung development, and become resident lung macrophages with auto-proliferation functions.

The lungs are exposed to the potentially harmful outside environment through the inhalation of gases, particles, suspended toxins, allergens, and pathogens. Accordingly, our immune system has evolved to provide protection against such insults without damaging the lung tissues. Mononuclear phagocytic (MNP) cells line the respiratory tract to process and clear potentially harmful inhaled substances, thereby securing the lung microenvironment. These cells are highly phagocytic in nature, produce large amounts of mediators, and recruit and activate the appropriate immune response to neutralize harmful insults. The majority of these MNP cells are macrophages (~90%) that are distributed throughout the airways, lung tissues, and alveolar tissues. Lung macrophages (LMs) either reside within the lungs or are recruited during lung insult. Resident macrophages self-replicate via a mechanism that is dependent on granulocyte-macrophage colony-stimulating factor (GM-CSF) and colony-stimulating factor-1 (CSF-1) signaling [1]. Therefore, questions have been raised regarding whether most lung tissue and airway macrophages are derived from bone marrow or bone marrow-derived blood monocytes, are recruited into lung tissues and airspaces, and differentiate with limited proliferating capability [2,3]. Recently, self-replicating and proliferating lung tissue-resident macrophages from embryonic [3] and fetal liver origins [4] have been described and characterized in a murine model [5] (Figure 1). These studies have led to the conceptualization of two distinct macrophage populations in the lungs: a tissue-resident population originating from the embryo or fetal liver in early life and a monocyte-derived population originating from hematopoietic stem cells and recruited from the blood. The phenotypic characteristics and functions of these two distinct macrophage populations are an area of active investigation.

## 2. Lung Macrophages (LM) Phenotypes

LMs have high levels of phenotypic and functional plasticity to adapt and respond to a variety of environmental insults. More than a decade ago, Mills and co-workers proposed a classification of macrophages to capture their phenotypic diversity, suggesting at least two distinct phenotypes, i.e., M1 and M2 macrophages [6]. M1 macrophages produce high levels of pro-inflammatory cytokines, have strong bactericidal properties, produce high levels of reactive nitrogen species and oxygen intermediates, promote a predominantly Th1 inflammatory milieu, and are referred to as “classically activated (pro-inflammatory) macrophages”. Induced by the exposure to Lipopolysaccharide (LPS) and IFN-γ, M1 macrophages secrete large amounts of pro-inflammatory cytokines, including IL-1β, TNF, and IL-12, and strongly express major histocompatibility complex (MHC) II (monocyte–macrophage marker), CD40, CD68, CD80, CD86, and iNOS. In contrast, M2 macrophages have been associated with a more Th2 inflammatory milieu, characterized by allergic responses and protection against parasites. Moreover, M2 macrophages are more immunomodulating in nature, are involved in tissue remodeling, including promoting macrophage efferocytic functions, and are referred to as “alternatively activated macrophages”. Induced by fungi, immunocomplexes, helminths, complement components, apoptotic cells, macrophage colony-stimulating factors, IL-4, and IL-13, M2 macrophages secrete anti-inflammatory cytokines, including IL-10, TGF-β, CCL18, and CCL22 [7], and are generally characterized by their high CD163 and CD206 expression levels [8].

This basic classification by Mills and co-workers has been further expanded by Mantovani and co-workers, dividing M2 macrophages into four different subtypes: M2a, M2b, M2c, and M2d [7]. Further phenotyping by Qian et al. identified tumor-associated macrophages [9] as well as those expressing T cell receptors and CD169. Currently, at least eight different distinct phenotypic populations have been identified [10]. Although the unique roles various M2 macrophages play remain unclear, the notion that macrophage plasticity is determined by the immediate microenvironment has been clearly supported.

## 3. LM Functional Responses

LMs are essential in regulating innate immune responses in the lungs. They are involved in cell defense against inhaled foreign matter (i.e., ambient irritants, pollutants, bacteria, and viruses), thereby keeping the airways clean. Following phagocytosis, LMs process all foreign materials engulfed and also act as antigen presenting cells, activating the adaptive immune response. These innate and adaptive immune responses are immunologically interdependent with LMs playing a pivotal role in the relationship between both responses. Following the processing of inhaled foreign matter, LMs generate and release mediators that stimulate the bone marrow, promoting the release of monocytes from the marrow, and enhance their recruitment into the lungs where they subsequently differentiate into tissue and airway macrophages [10], further supplementing resident LMs.

LM responses to engulfed foreign matter could somewhat vary depending on the nature of the particles, specifically material size, geometry, and surface characteristics. LMs comfortably phagocytose smaller particles (<10 μm) [11] but frequently collectively cooperate and form giant multinucleated cells to process larger ones (>10 μm) [12]. Champion and Mitragotri [13] showed that particle shape could also impact the ease of LM phagocytosis. In addition, particles with uneven and rougher surfaces promote cell adherence and modulate LM production of pro-inflammatory cytokines and chemokines [14]. Lastly, organic foreign materials, such as microorganisms, require opsonization to promote Fc receptor-mediated phagocytosis and clearance [15] or interact with toll-like receptors that recognize both Gram-positive and Gram-negative bacteria for phagocytosis [16,17].

## 4. Macrophages in Chronic Obstructive Pulmonary Disease (COPD)

COPD is an inflammatory disease of the lungs that is characterized by air flow limitations that are not fully reversible. It is characterized by clinical symptoms such as cough, sputum production, and shortness of breath with episodes of exacerbation, especially during the late stages of the disease. The disease is progressive in nature and the World Health Organization has estimated that 3.17 million deaths were caused by the COPD in 2015. Although cigarette smoking is still the major cause of COPD in developed countries, biomass exposure is a more significant cause of COPD in developing countries [18]. For both exposure types, LMs play a central role in processing and clearing particles from the lungs. Given the chronicity of these exposures, LMs’ downstream functional responses thereto participate in the development of COPD [19]. A small fraction of COPD are caused by a genetic deficiency in α-1 antitrypsin, but long-term exposure to air pollutants and occupational exposures, such as dust particles, fumes, and chemicals, are more important on a worldwide scale [20]. The significantly increased number of macrophages in the sputum and Bronchoalveolar Lavage Fluid (BALF) of patients with COPD support this notion [21,22]. In COPD, LMs also secrete large amounts of potentially tissue-damaging enzymes, such as elastase, metalloproteinase (MMP)-2, MMP-9, MMP-12, and cathepsin S, in response to foreign particulate matter and microorganism exposure [22,23]. A majority of acute COPD exacerbations are triggered by either viral or bacterial respiratory infections [24] that could lead to bacterial colonization of the airways, subsequently contributing to frequent exacerbations [25]. In addition, continuous exposure to cigarette smoke or biomass markedly depletes intracellular anti-oxidants, such as glutathione, causing excessive oxidative stress, which suppresses LMs’ bacterial phagocytic and efferocytic functions [26]. Therefore, LMs in COPD generate a more pro-inflammatory milieu that could cause tissue damage, as well as defective immune surveillance and protective (phagocytic) functions that collectively contribute to the progression of COPD.

## 5. LMs and Bacterial Colonization of COPD Airways

The lung airways have their own distinct microbiome as revealed by recent next-generation sequencing technologies, including 16 s RNA gene measurement [20]. Studies in individuals with COPD have revealed alterations in the microbiome that vary with the severity of COPD, acute COPD exacerbations, and use of steroids and/or antibiotics [27]. Changes in the lung microbiome may contribute to the pathogenesis of COPD by influencing inflammatory and/or immune processes in the lungs. Although LMs play a central role in clearing harmful bacteria from the lungs, this function deteriorates as the disease progresses. Berenson et al. reported that the severity of COPD (FEV 1% predicted) correlated with impaired LM phagocytosis for nontypeable *Haemophilus influenzae* (NTHi) and *Moraxella catarrhalis* [28]. Moreover, studies using clone library analysis of the 16 s RNA gene sequence have reported that *H. influenzae* and *M. catarrhalis* were more frequently identified as causative bacteria for pneumonia and/or exacerbations of COPD as well as disease progression [29]. Similarly, *Streptococcus pneumoniae*, which also causes acute COPD exacerbations, had been associated with the progression of emphysema possibly by inducing LMs to produce MMP-12 [30]. Therefore, the defective phagocytic and efferocytic functions of LMs in COPD could contribute to the colonization of the airways with various bacteria, specifically those known to cause acute exacerbations and pneumonia during COPD. Consequently, these infections have been related to the progression of COPD [29].

A pivotal function of LMs is to remove and clear cell debris and dead or damaged cells following an inflammatory insult to the lungs. Such a process, which is termed efferocytosis, becomes defective in subjects with COPD [31]. Accordingly, sputum and airway neutrophils are elevated in most subjects with COPD and further increase during acute COPD exacerbations [32]. The defective clearance of these recruited neutrophils allows for the accumulation of necrotic neutrophils that indiscriminately release toxic granule proteins containing neutrophil elastase, a protease that has been associated with tissue damage and COPD progression [33]. The recognition of activated neutrophil extracellular trap-like structures containing their DNA and granule proteins, such as histones and neutrophil elastase [34], is imperative for the proper clearance of cell debris following an inflammatory insult to the lungs. Neutrophil elastase degrades extracellular matrix components as well as lung elastic fibers related to the development of pulmonary emphysema, and promotes the release of mucin via an epidermal growth factor receptor-dependent mechanism [35]. Considering that LMs are the primary “janitors” of the lungs, dysfunctional processing and clearance of apoptotic and necrotic cells and cell debris could clearly contribute to ongoing lung tissue inflammation in subjects with COPD, even long after they stop smoking [36].

## 6. Macrophage Phenotypes in COPD

**Figure 2:**
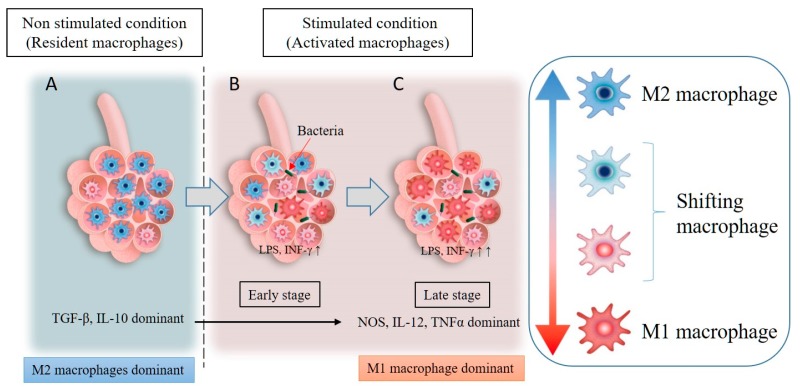
Resident macrophages are mainly of the M2 phenotype (M2 dominant) under non-stimulated conditions and secrete anti-inflammatory cytokines (TGF-β, IL-10) (**A**). An inflammatory stimulus in the lungs, such as exposure to pathogens, LPS, particulate matter, or INF-γ, leads to the phenotype switching of resident macrophages from M2 to M1 (**B**). In addition, circulating monocytes will be recruited into the lung tissues and activated by the inflammatory stimulus, with the majority of the macrophages in the inflammatory zone being M1 macrophages (M1 dominant). These M1 macrophages secrete inflammatory cytokines, such as NOS, IL-12, and TNF-α (**C**).

**Figure 3:**
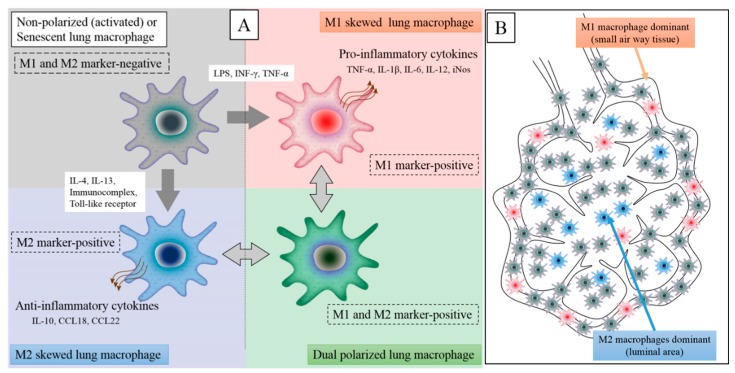
Patients with COPD have at least four types of macrophages in their lung tissues. Non-polarized or senescent macrophages are M1 and M2 marker-negative and secrete low levels of inflammatory and anti-inflammatory cytokines (**left** upper). Upon stimulation by Th1 cytokines (e.g., LPS and/or INF-γ), these cell become polarized to predominantly M1 lung macrophages and secrete high levels of pro-inflammatory cytokines (**right** upper). However, when non-polarized macrophages are stimulated by Th2 cytokines (e.g., IL-4 and/or IL-13), they become polarized to predominantly M2 lung macrophages and secrete high levels of level anti-inflammatory cytokines (left lower). COPD lung tissues also contain a large population of dual-positive macrophages (M1 and M2 marker-positive) (right lower). Nonetheless, the functional properties of dual-positive macrophages are still unclear (**A**). In COPD lung tissues, M1 macrophages are predominantly found in small airway wall tissues and M2 macrophages in airspaces while abundant non-polarized macrophages are present in both compartments. The distribution of dual-positive macrophages is still unclear (**B**).

The role of distinct macrophage phenotypes (M1 versus M2) in COPD is unclear. Environmental exposures causing COPD produce either an acute or chronic insult resulting in an inflammatory response in the lung tissues that promotes both M1 (pro-inflammatory) and M2 (reparative) macrophages. An example would be the simultaneous elevation of iNOS (an M1 marker) and arginase (an M2 marker) activity in COPD tissue [37]. Furthermore, non-polarized macrophages that are negative for both M1 and M2 markers or dual positive for both markers have been reported by several investigators [37]. Non-polarized macrophages are more frequently distributed in normal lung tissues, while dual positive (both M1 and M2 marker-positive) macrophages are more frequently distributed in lung tissues of subjects with severe COPD [37]. The presence of COPD suggests that these macrophages have been recently recruited (not resident) and both classically (M1) and alternatively (M2) activated through the expression of markers for both phenotypes. Both M1 and M2 macrophages have been reported in smokers with and without COPD and other lung diseases, such as sarcoidosis [38]. Nonetheless, the plasticity of macrophages to change phenotypes largely depends on their microenvironment [39] (Figure 2). Using a monocyte cell line (THP-1 cells), Genin and co-workers generated macrophages through incubation with phorbol 12-myristate 13-acetate. The macrophages they obtained did not possess any of the M1 or M2 activation markers (M0 macrophages). By incubating these M0 macrophages with specific phenotypic mediators (see Figure 2), they were able to obtain differentiated and polarized M1 and M2 macrophages [40]. The complexity of the COPD microenvironment is such that four distinct phenotypes of macrophages can be clearly identified (Figure 3A): non-polarized macrophages (both M1 and M2 marker-negative), M1-skewed macrophages (strong M1 marker-positive), M2-skewed macrophages (strong M2 marker-positive), and hybrid macrophages (both M1 and M2 marker-positive or dual positive). Little is known about the distribution of these different phenotypes of macrophages in the tissues and airspaces in the COPD lung. Recently, Eapen and co-workers showed that non-polarized macrophages were present in both small airway tissues and luminal airspaces, whereas M1 macrophages predominate in small airways and M2 macrophages predominate in luminal areas in COPD lung tissues compared to normal control [41] (Figure 3B). Owing to chronic and ongoing insult (cigarette smoke and colonization/infection), individuals with COPD experience constant changes to their lung environment, potentially influencing macrophage phenotype with time. Accordingly, elucidating the origin and functional properties of these four LM phenotypes could pave the way for the development of more macrophage-specific therapeutic interventions for COPD.

## 7. Phagocytic and Efferocytic Functions of Macrophages in COPD

Inhaled foreign substances, such as bacteria, fungi, viruses, pollen, and air pollutants, are processed and cleared from the lungs to maintain homeostasis. During COPD, the total number of airway macrophages (BAL) are significantly increased, while phagocytosis and elimination of microorganisms and apoptotic cells are paradoxically impaired [42], suggesting defective functional properties of the macrophages. Several investigators have reported impairments in LMs’ ability to phagocytose fungi, bacteria, and air pollution (in a study using latex beads) (compared to normal controls) [28], while patients with COPD also displayed impaired LM efferocytosis [43]. Furthermore, peripheral blood monocytes from patients with COPD were significantly less effective in killing *Candida albicans* than those from control subjects [44,45], suggesting the presence of these defects even before recruitment into lung tissues. In a rat model of COPD, macrophage phagocytosis of Aspergillus had been impaired [46]. Moreover, LMs from donors with COPD had a greater impairment in phagocytic activity against NTHi compared to those from donors without COPD [47,48]. Macrophages derived from blood monocytes of patients with COPD have reduced phagocytic activity against *H. influenzae* and *S. pneumoniae* [49]. However, COPD macrophages showed no defect in the non-specific phagocytosis of latex beads [43,49], suggesting a defect in receptor- or opsonization-mediated phagocytosis. This notion has been supported by studies using rhinovirus exposure to impair LM phagocytosis and immune responses to bacterial products [50]. Clearly, the mechanisms of LM phagocytic dysfunction remain unclear and need further study.

Efferocytosis, a process by which dead and apoptotic cells are removed from the body, is an essential function for maintaining a healthy lung microenvironment. Various studies have reported impairments in the efferocytic function of LMs among patients with COPD [42]. A significantly greater impairment in efferocytosis had been reported in patients with non-eosinophilic asthma or COPD compared to those with eosinophilic asthma [32]. Although the underlying mechanism for this disorder has yet to be fully elucidated, several hypotheses have been proposed. A cigarette-smoking model revealed that apoptotic cell clearance had been impaired through oxidant-dependent activation of RhoA [51]. Furthermore, galectin-3, an S-type lectin known to regulate the phenotype and function of macrophages, is significantly decreased in the BAL of patients with COPD [52]. Lastly, Hodge et al. reported that reduced LM efferocytosis in patients with COPD was associated with a reduced expression of M2 mannose receptors (CD206) and several other key macrophage recognition molecules [42], suggesting a direct link between macrophage functional properties and phenotype. Defective efferocytosis allows for cytotoxic products of dead or dying cells to be released, possibly leading to lung tissue damage and COPD progression.

## 8. Therapeutic Targeting of Macrophages in COPD

Currently, no pharmacological COPD intervention has been shown to significantly slow the decline in FEV1 over time. Although bronchodilators have been the primary treatment option for airflow limitations typical in COPD [24], they predominantly address the symptoms and not the underlying immune pathogenesis of the disease. Novel treatments target specific molecular pathways thought to be pivotal in the pathogenesis of the disease, such as small molecule inhibitors with anti-inflammatory activity against p38 mitogen-activated protein kinases, phosphatidyl-inositol-3 kinase, and Rho kinase [53].

Hodge et al. reported that azithromycin increases the phagocytosis of apoptotic bronchial epithelial cells [54] and improves the phagocytosis of bacteria through both alveolar- and monocyte-derived macrophages in patients with COPD [55,56]. Macrolides with diminished antibiotic activity (non-antibiotic macrolides) significantly improved the phagocytosis of apoptotic cells and NTHi, an approach that could potentially reduce airway inflammation in patients with chronic inflammatory lung conditions, such as COPD and bronchiectasis [57]. In a recent study, azithromycin 250 mg daily for 8 weeks altered both lung microbiota and metabolome in patients with COPD [58]. According to these reports, macrolides such as azithromycin might contribute to COPD treatment by changing the lung microbiome via macrophage phagocytic function.

Several epidemiological studies have shown that inhaled corticosteroids increase the incidence of pneumonia [59,60], tuberculosis, and upper respiratory tract infections [61] in patients with COPD. Mouse models have shown that fluticasone treatment in the presence of apoptotic cells significantly reduced in vitro LM killing of pneumococci in part by delaying phagolysosome acidification without affecting the production of reactive oxygen or nitrogen species [62]. Inhaled fluticasone propionate has also been shown to impair pulmonary clearance of *Klebsiella pneumoniae* [63]. Treatment with lovastatin [64], low-dose oxygen [65], and the acute phase reactant α-1 antitrypsin [66] increased the efferocytosis function of LMs in a mouse model. Moreover, several studies have shown that statins beneficially impact innate immune responses in the lungs, including in subjects with COPD [67]. They also reduce airway macrophages and pro-inflammatory mediators, such as IL-17, and increase anti-inflammatory mediators, such as IL-10 [68].

Collectively, these studies suggest that altering the recruitment and functional properties of LMs could potentially reduce ongoing inflammatory responses in lung tissues of subjects with COPD.

Macrophage efferocytosis of eosinophils is impaired in patients with COPD, the degree of which is related to the severity and frequency of COPD exacerbations [69]. In human alveolar macrophages from patients with COPD [70] and in LMs of mice exposed to cigarette smoke [71], d-series resolvins reduced the levels of pro-inflammatory cytokines produced and enhanced phagocytic function, suggesting that cigarette smoke exposure impairs efferocytosis via the inhibition of the HDAC/Rac/CD9 pathways [72]. Phosphodiesterase (PDE) inhibitors, such as aminophylline/theophylline, are effective in restoring efferocytosis impairment in LMs, while recent studies using roflumilast [73], a newer PDE-4 inhibitor, revealed alterations in the LM phenotype to a more reparative M2 type [74]. These studies underscore the known anti-inflammatory properties of PDE inhibitors, suggesting that such effects are produced via impacting LM function by promoting their anti-inflammatory or reparative functions [75].

In a mouse model, vitamin D-deficient mice exhibited an accelerated decline in lung function and a greatly impaired ex vivo phagocytic and oxidative burst function of alveolar macrophages after cigarette smoke exposure compared to control mice [76]. Accordingly, the administration of bisphosphonate alendronate via aerosol inhalation in such mice not only ameliorated elastase-induced emphysema by inhibiting macrophage migratory and phagocytic activities but also blunted the inflammatory response of alveolar macrophages by inhibiting nuclear factor-κB signaling [77]. Lastly, oxidative stress reduces the levels of macrophage phagocytic activity in COPD [78]. Oxidized phospholipids generated through chronic cigarette smoke exposure play a crucial role in inhibiting the phagocytic function of LMs, thus impairing the innate pulmonary anti-bacterial defenses in mice exposed to cigarette smoke [79]. Therapeutic approaches that augment pulmonary antioxidant defenses could be beneficial for reducing the oxidative stress-driven impairment in LM phagocytosis among smokers and patients with COPD [80,81].

## 9. Conclusions

LMs are essential innate immune effector cells that elicit and control inflammatory responses in the lungs after exposure to environmental pollutants, including cigarette smoke: the main environmental risk factor for COPD. Although LMs are pivotal for processing and removing inhaled irritants from the lungs, they could also inflict damage to lung tissues in the process by promoting a dysregulated inflammatory response, which may in turn lead to dysfunctional tissue repair and a persistent state of chronic low-grade inflammation in the lungs. Despite being poorly understood, the immune pathogenic mechanisms by which COPD develops and progresses need to be elucidated in order to manage and treat the disease. Given that LMs are essential effector cells in this process, we herein describe the characteristics and behavior of macrophages in COPD. Studies that unravel the mechanisms promoting macrophage anti-inflammatory and reparative functions could contribute to the development of more targeted therapeutic interventions to reduce the destructive inflammatory response induced by cigarette smoke and environmental exposures that eventually lead to COPD.
